# Multifunctional Roles of Plant Dehydrins in Response to Environmental Stresses

**DOI:** 10.3389/fpls.2017.01018

**Published:** 2017-06-09

**Authors:** Yang Liu, Qiping Song, Daxing Li, Xinghong Yang, Dequan Li

**Affiliations:** State Key Laboratory of Crop Biology, Shandong Key Laboratory of Crop Biology, College of Life Sciences, Shandong Agricultural UniversityTai’an, China

**Keywords:** dehydrin, conserved segments, LEA protein, abiotic stresses, function

## Abstract

To respond to environmental changes, plants have developed complex mechanisms that allow them to rapidly perceive and respond to abiotic stresses. Late embryogenesis abundant (LEA) proteins are a large and diverse family that play important roles in environmental stress tolerance in plants. Dehydrins belong to group II LEA proteins, which are considered stress proteins involved in the formation of plants’ protective reactions to dehydration. Some studies have demonstrated that dehydrins could binding metal ions or lipid vesicles. *In vitro* experiments revealed that dehydrins could protect the activity of enzyme from damage caused by environmental stress. Although many studies have been conducted to understand their roles in abiotic stresses, the molecular function of dehydrins is still unclear. In this review, to generate new ideas for elucidating dehydrins’ functions, we highlight the functional characteristics of dehydrins to understand their roles under environmental stress in plants.

## Introduction

Late embryogenesis abundant (LEA) proteins are related with the acquisition of desiccation tolerance in plants and are accumulated during the late stages of seed development (Dure and Galau, 1981; Liu et al., 2013, 2016). According to the conserved segments, LEA proteins can be categorized into seven different groups (Battaglia et al., 2008). Dehydrins belong to the group II LEA proteins, which were initially identified as the “D-11" family in the developing cotton embryos. Dehydrins are considered stress proteins involved in the formation of plants’ protective reactions to dehydration. They can also be considered as hydrophilins. They are highly hydrophilic, containing a high proportion of charged and polar amino acids and a low fraction of hydrophobic, non-polar residues, and lack tryptophan and cysteine residues. Based on the presence of conserved sequences (Y-, S-, and K-segments), dehydrins were classified into the different sub-classes YnKn, YnSKn, KnS, SKn and Kn (Garay-Arroyo et al., 2000).

Although the function of dehydrins has not been clearly understood, many studies have demonstrated dehydrins play important roles in abiotic stress tolerance. Overexpression of the *Prunus mume* dehydrin *PmLEAs* in tobacco and *Escherichia coli* enhances tolerance to cold and drought (Bao et al., 2017). Overexpression of the *Solanum habrochaites* dehydrins *ShDHN* enhances transgenic tomato tolerance to multiple abiotic stresses (Liu et al., 2015). Most dehydrins are distributed along with several ubiquitous dehydration-stress response protein types in plants (Bao et al., 2017; Fan et al., 2017). However, some dehydrins were also found distributed in the vegetative tissues of plants grown under normal conditions, which suggested that the dehydrins may play important roles in the plant growth. Recently, studies indicate that the *Medicago truncatula* Y_2_K_4_-type dehydrin MtCAS31 could interact with AtICE1 (an inducer of CBF expression 1). The interaction of AtICE1with MtCAS31 plays important roles in stomatal development, enhancing the drought resistance by decreasing in stomatal density of transgenic *Arabidopsis thaliana* (Xie et al., 2012).

## The Roles of the Different Conserved Segments in Dehydrins

The K-segment can form an amphiphilic α-helix, which representing a highly conserved 15 amino acid segment (EKKGIMDKIKEKLPG) that has been found in all dehydrins (Malik et al., 2017). The α-helix of dehydrins may help stabilize the proteins and the cellular membranes under environmental stress conditions. The *Citrus unshiu* K_3_S-type dehydrin CuCOR19 was found to form an α-helix in the presence of SDS (sodium dodecyl sulfate) (Hara et al., 2001). The K-segment of the maize dehydrin DHN1 is essential for binding to the anionic phospholipid vesicles, and the adoption of α-helicity by the K-segment accounts for the conformational change in dehydrins upon binding to SDS or anionic phospholipid vesicles (Koag et al., 2009). The K-segment of wheat dehydrin DHN-5 is required for the protection of β-glucosidase and LDH (lactate dehydrogenase) activities *in vitro* (Drira et al., 2013). The lysine-rich segment of the *Craterostigma plantagineum* disordered stress protein CDeT11-24 is important for the protection of proteins from damage caused by water stress and the phosphatidic acid (PA) binding (Petersen et al., 2012). The cold-induced dehydrin Lti30 can bind to cellular membranes via its K-segments, which locally fold into α-helix on the cellular membrane surface. The protein Lti30’s K-segments electrostatically trap the negatively charged lipid head (Eriksson et al., 2011, 2016).

The Y-segment, representing a conserved segment [V/T]D[E/Q]YGNP, is usually found in various tandem copies in the N-terminus of dehydrins (Campbell and Close, 1997). The Y-segment of dehydrins has sequence similarity to the nucleotide-binding site of plant and bacterial chaperones [(V/T) DEYGNP]. However, there is no experimental evidence suggesting that the Y-segment binds nucleotides (Close, 1996; Hughes et al., 2013).

Some dehydrins contain the S-segment, which consists of a tract of Ser residues and can be phosphorylated. The phosphorylation of the S-segment is suggested to promote dehydrins interaction with the specific signal peptides followed by their translocation into the nucleus (Goday et al., 1994; Close, 1996; Jiang and Wang, 2004). The S-segment can be phosphorylated by protein kinase, which may influence the location and the ability to bind metal ions (Goday et al., 1994; Alsheikh et al., 2003). The three amino acid residues EDD of the maize dehydrin Rab17 are the CKII phosphorylation recognition site (Jensen et al., 1998).

The Φ-segments are less conserved motifs, which lay interspersed between K-segments (Campbell and Close, 1997).

## The Function of Dehydrins

### Binding Metal Ions

Under control conditions, catalytic metal ions (such as Cu^2+^ and Zn^2+^) mainly exist as metal-protein complexes in plants. Nevertheless, they can be released as free ions when plants are subjected to stress conditions. These free ions are required to produce ROS (reactive oxygen species) via the Fenton or the Haber–Weiss reactions (Mittler, 2002). It has been reported that the *Ricinus* KS-type dehydrin ITP is the first member of the LEA protein family shown to be active in the long-distance transport micronutrients (Krüger et al., 2002). The *Arabidopsis thaliana* dehydrin AtHIRD11 and citrus dehydrin CuCOR15 can bind the metal ions Fe^3+^, Co^2+^, Ni^2+^, Cu^2+^ and Zn^2+^ over Mg^2+^and Ca^2+^ (Hara et al., 2005, 2011).

### Binding DNA

The bioinformatic analysis of the “Protein or Oligonucleotide Probability Profile (POPP)” predicted that dehydrins could bind DNA (Wise and Tunnacliffe, 2004). Until now, several studies have reported that LEA protein can bind DNA. Citrus dehydrin CuCOR15 can bind nucleic acids, a process that depends on zinc ions. The histidine-rich domain (TTDVHHQQQYHGGEH) and the lysine-rich domain (GGEGAHGEEKKKKKKEKKK) were the DNA-binding domains (Hara et al., 2009). Y_2_K-type dehydrin VrDhn1 exhibited a low affinity for non-specific interaction with DNA, and the exogenous addition metal ions of Zn^2+^ or Ni^2+^ stimulated the interaction (Lin et al., 2012).

### Binding Phospholipid

Phosphatidic acid is the important stress-signaling phospholipid, and its accumulation is triggered in response to various environmental stress conditions, such as salinity, drought and cold (Frank et al., 2000; Katagiri et al., 2005). The PA concentration in the turgescent plant membranes is only approximately 1%, but increases under water deficit stress. The formation of PA depends on the activity of phospholipase D, which can be induced by ABA and dehydration (Katagiri et al., 2001).

Many studies have demonstrated that dehydrin can bind with phospholipids, and the binding was not limited to a certain type of phospholipids (Kooijman et al., 2007; Koag et al., 2009; Petersen et al., 2012). The maize SK_2_- type dehydrin DHN1 can bind PA. Although most of the DHN1 protein is disorder, the K-segment can adopt α-helical conformation. An electrostatic nature of the interaction has been postulated. The basic amino acids, such as arginine and lysine, are required for the interaction in the binding domain (Kooijman et al., 2007; Koag et al., 2009). The Kn-type dehydrin Lti30 can interact electrostatically with vesicles of both negatively charged phospholipids (phosphatidic acid, phosphatidyl serine and phosphatidyl glycerol) and zwitterionic (phosphatidyl choline). The Lti30 interaction of lipid is regulated by phosphorylation, pH dependent His on/off switch, and the reversal of membrane binding by proteolytic digest (Eriksson et al., 2011).

## Protecting the Activity of Proteins

The hypothesis that dehydrin can bind proteins to prevent its denaturation has been suggested for many years. Many studies have demonstrated that dehydrin can protect the activity of LDH and malate dehydrogenase from damage caused by various environmental stresses (Drira et al., 2013, 2015; Yang et al., 2015). The KS-type dehydrin AtHIRD11 recovers LDH activity inhibited by copper with the contribution of histidine residues (Hara et al., 2016). However, the interaction between the dehydrins and the protected proteins has been not found.

## Scavenging the Reactive Oxygen Species

Many studies have indicated that dehydrins can directly scavenge the free radicals. The citrus dehydrin CuCOR19 displays a stronger inhibition against free radical-inducing peroxidation. The overexpression CuCOR19 in transgenic tobacco can inhibit lipid peroxidation under cold stress (Hara et al., 2003). The amino acids, such as histidine, lysine and glycine, are targets for radical-mediated oxidation (Dean et al., 1997). The content of the three amino acids is high in mostly dehydrins, which can reduce the level of ROS. The *Arabidopsis thaliana* KS-type dehydrin AtHIRD11 can reduce ROS generation from copper. The length of the peptides and the histidine contents are fundamental factors that can influence the ROS decrease by the KS-type dehydrins (Hara et al., 2013, 2016).

## Conclusion

To summarize the data presented above, the functional mechanisms are listed as follows.

(I) Dehydrins can bind metal ions, which can inhibit the production of ROS at the source.
(II) The high content of the antioxidant amino acids, such as lysine, histidine and glycine, can scavenge ROS through oxidative modification.
(III) Dehydrins can non-specifically bind proteins and membranes, which can protect the function and structure of the protein or membrane from damage caused by environmental stresses.
(Iv) Dehydrins can bind DNA, which may repair or protect the DNA from damage caused by environmental stresses.

## Author Contributions

YL wrote the article. QS and DaL gave positive suggestion about this article. DeL and XY revised the paper and gave positive suggestion. All authors read and approved the manuscript.

## Conflict of Interest Statement

The authors declare that the research was conducted in the absence of any commercial or financial relationships that could be construed as a potential conflict of interest.

## References

[B1] Alsheikh M. K. Heyen B. J. Randall S. K. (2003). Ion binding properties of the dehydrin ERD14 are dependent upon phosphorylation. *J. Biol. Chem.* 278 40882–40889. 10.1074/jbc.M307151200 12917402

[B2] Bao F. Du D. An Y. Yang W. Wang J. Cheng T. (2017). Overexpression of *Prunus mume* dehydrin genes in tobacco enhances tolerance to cold and drought. *Front. Plant Sci.* 8:151 10.3389/fpls.2017.00151 PMC5293821 28224001

[B3] Battaglia M. Olvera-Carrillo Y. Garciarrubio A. Campos F. Covarrubias A. A. (2008). The enigmatic LEA proteins and other hydrophilins. *Plant Physiol.* 148 6–24. 10.1104/pp.108.120725 18772351 PMC2528095

[B4] Campbell S. A. Close T. J. (1997). Dehydrins: genes, proteins, and associations with phenotypic traits. *New Phytol.* 137 61–74. 10.1046/j.1469-8137.1997.00831.x

[B5] Close T. J. (1996). Dehydrins: emergence of a biochemical role of a family of plant dehydration proteins. *Physiol. Plant.* 97 795–803. 10.1111/j.1399-3054.1996.tb00546.x

[B6] Dean R. T. Fu S. Stocker R. Davies M. J. (1997). Biochemistry and pathology of radical-mediated protein oxidation. *Biochem. J.* 324 1–18. 10.1042/bj3240001 9164834 PMC1218394

[B7] Drira M. Saibi W. Amara I. Masmoudi K. Hanin M. Brini F. (2015). Wheat dehydrin K-segments ensure bacterial stress tolerance, antiaggregation and antimicrobial effects. *Appl. Biochem. Biotechnol.* 175 3310–3321. 10.3389/fpls.2015.00406 25637507

[B8] Drira M. Saibi W. Brini F. Gargouri A. Masmoudi K. Hanin M. (2013). The K-segments of the wheat dehydrin DHN-5 are essential for the protection of lactate dehydrogenase and β-glucosidase activities in vitro. *Mol. Biotechnol.* 54 643–650. 10.1007/s12033-012-9606-8 23054631

[B9] Dure L. Galau G. A. (1981). Developmental biochemistry of cotton seed embryogenesis and germination XIII. Regulation of biosynthesis of principal storage proteins. *Plant Physiol.* 68 187–194. 10.1104/pp.68.1.187 16661868 PMC425913

[B10] Eriksson S. Eremina N. Barth A. Danielsson J. Harryson P. (2016). Membrane-induced folding of the plant-stress protein Lti30. *Plant Physiol.* 71 932–943. 10.1104/pp.15.01531 PMC4902575 27208263

[B11] Eriksson S. K. Kutzer M. Procek J. Grobner G. Harryson P. (2011). Tunable membrane binding of the intrinsically disordered dehydrin Lti30, a cold-induced plant stress protein. *Plant Cell* 23 2391–2404. 10.1105/tpc.111.085183 21665998 PMC3160030

[B12] Fan N. Lv A. Xie J. Yuan S. An Y. Zhou P. (2017). Expression of CdDHN4, a novel YSK2-type dehydrin gene from bermudagrass, responses to drought stress through ABA-dependent signal pathway. *Front. Plant Sci.* 8:748 10.3389/fpls.2017.00748 PMC5433092 28559903

[B13] Frank W. Munnik T. Kerkmann K. Salamini F. Bartels D. (2000). Water deficit triggers phospholipase D activity in the resurrection plant *Craterostigma plantagineum*. *Plant Cell* 12 111–123. 10.1105/tpc.12.1.111 10634911 PMC140218

[B14] Garay-Arroyo A. Colmenero-Flores J. M. Garciarrubio A. Covarrubias A. A. (2000). Highly hydrophilic proteins in prokaryotes and eukaryotes are common during conditions of water deficit. *J. Biol. Chem.* 275 5668–5674. 10.1074/jbc.275.8.5668 10681550

[B15] Goday A. Jensen A. B. Culiáñez-Macià F. Albà M. M. Figueras M. Serratosa J. (1994). The maize abscisic-acid responsive protein Rab17 is located in the nucleus and interacts with nuclear localization signals. *Plant Cell* 6 351–360. 10.1105/tpc.6.3.351 8180497 PMC160438

[B16] Hara M. Fujinaga M. Kuboi T. (2005). Metal binding by citrus dehydrin with histidine-rich domains. *J. Exp. Bot.* 56 2695–2703. 10.1093/jxb/eri262 16131509

[B17] Hara M. Kondo M. Kato T. (2013). A KS-type dehydrin and its related domains reduce Cu-promoted radical generation and the histidine residues contribute to the radical-reducing activities. *J. Exp. Bot.* 64 1615–1624. 10.1093/jxb/ert016 23382551 PMC3617826

[B18] Hara M. Monna S. Murata T. Nakano T. Amano S. Nachbar M. (2016). The Arabidopsis KS-type dehydrin recovers lactate dehydrogenase activity inhibited by copper with the contribution of his residues. *Plant Sci.* 245 135–142. 10.1016/j.plantsci.2016.02.006 26940498

[B19] Hara M. Shinoda Y. Kubo M. Kashima D. Takahashi I. Kato T. (2011). Biochemical characterization of the *Arabidopsis* KS-type dehydrin protein, whose gene expression is constitutively abundant rather than stress dependent. *Acta Physiol. Plant.* 33 2103–2116. 10.1007/s11738-011-0749-1

[B20] Hara M. Shinoda Y. Tanaka Y. Kuboi T. (2009). DNA binding of citrus dehydrin promoted by zinc ion. *Plant Cell Environ.* 32 532–541. 10.1111/j.1365-3040.2009.01947.x 19183287

[B21] Hara M. Terashima S. Fukaya T. Kuboi T. (2003). Enhancement of cold tolerance and inhibition of lipid peroxidation by citrus dehydrin in transgenic tobacco. *Planta* 217 290–298. 10.1007/s00425-003-0986-7 12783337

[B22] Hara M. Terashima S. Kuboi T. (2001). Characterization and cryoprotective activity of cold-responsive dehydrin from *Citrus unshiu*. *J. Plant Physiol.* 158 1333–1339. 10.1078/0176-1617-00600

[B23] Hughes S. L. Schart V. Malcolmson J. Hogarth K. A. Martynowicz D. M. Tralman-Baker E. (2013). The importance of size and disorder in the cryoprotective effects of dehydrins. *Plant Physiol.* 163 1376–1386. 10.1104/pp.113.226803 24047864 PMC3813657

[B24] Jensen A. B. Goday A. Figueras M. Jessop A. C. Pages M. (1998). Phosphorylation mediates the nuclear targeting of the maize Rab17 protein. *Plant J.* 13 691–697. 10.1046/j.1365-313X.1998.00069.x 9681011

[B25] Jiang X. Wang Y. (2004). β-Elimination coupled with tandem mass spectrometry for the identification of in vivo and in vitro phosphorylation sites in maize dehydrin DHN1 protein. *Biochemistry* 43 15567–15576. 10.1021/bi0483965 15581369

[B26] Katagiri T. Ishiyama K. Kato T. Tabata S. Kobayashi M. Shinozaki K. (2005). An important role of phosphatidic acid in ABA signaling during germination in *Arabidopsis thaliana*. *Plant J.* 43 107–117. 10.1111/j.1365-313X.2005.02431.x 15960620

[B27] Katagiri T. Takahashi S. Shinozaki K. (2001). Involvement of a novel *Arabidopsis* phospholipase D, AtPLDδ, in dehydration-inducible accumulation of phosphatidic acid in stress signalling. *Plant J.* 26 595–605. 10.1046/j.1365-313x.2001.01060.x 11489173

[B28] Koag M. C. Wilkens S. Fenton R. D. Resnik J. Vo E. Close T. J. (2009). The K-segment of maize DHN1 mediates binding to anionic phospholipid vesicles and concomitant structural changes. *Plant Physiol.* 150 1503–1514. 10.1104/pp.109.136697 19439573 PMC2705017

[B29] Kooijman E. E. Tieleman D. P. Testerink C. Munnik T. Rijkers D. T. Burger K. N. (2007). An electrostatic/hydrogen bond switch as the basis for the specific interaction of phosphatidic acid with proteins. *J. Biol. Chem.* 282 11356–11364. 10.1074/jbc.M609737200 17277311

[B30] Krüger C. Berkowitz O. Stephan U. W. Hell R. (2002). A metalbinding member of the late embryogenesis abundant protein family transports iron in the phloem of *Ricinus communis* L. *J. Biol. Chem.* 277 25062–25069. 10.1074/jbc.M201896200 11983700

[B31] Lin C. H. Peng P. H. Ko C. Y. Markhart A. H. Lin T. Y. (2012). Characterization of a novel Y2K-type dehydrin VrDhn1 from *Vigna radiata*. *Plant Cell Physiol.* 53 930–942. 10.1093/pcp/pcs040 22440330

[B32] Liu H. Yu C. Li H. Ouyang B. Wang T. Zhang J. (2015). Overexpression of ShDHN, a dehydrin gene from *Solanum habrochaites* enhances tolerance to multiple abiotic stresses in tomato. *Plant Sci.* 231 198–211. 10.1016/j.plantsci.2014.12.006 25576005

[B33] Liu Y. Liang J. Sun L. Yang X. Li D. (2016). Group 3 LEA protein, ZmLEA3, is involved in protection from low temperature stress. *Front. Plant Sci.* 7:1011 10.3389/fpls.2016.01011 PMC4944394 27471509

[B34] Liu Y. Wang L. Xing X. Sun L. Pan J. Kong X. (2013). ZmLEA3, a multifunctional group 3 LEA protein from maize (*Zea mays* L.), is involved in biotic and abiotic stresses. *Plant Cell Physiol.* 54 944–959. 10.1093/pcp/pct047 23543751

[B35] Malik A. A. Veltri M. Boddington K. F. Singh K. K. Graether S. P. (2017). Genome analysis of conserved dehydrin motifs in vascular plants. *Front. Plant Sci.* 8:709 10.3389/fpls.2017.00709 PMC5415607 28523013

[B36] Mittler R. (2002). Oxidative stress, antioxidants and stress tolerance. *Trends Plant Sci.* 7 405–410. 10.1016/S1360-1385(02)02312-9 12234732

[B37] Petersen J. Eriksson S. K. Harryson P. Pierog S. Colby T. Bartels D. (2012). The lysine-rich motif of intrinsically disordered stress protein CDeT11-24 from *Craterostigma plantagineum* is responsible for phosphatidic acid binding and protection of enzymes from damaging effects caused by desiccation. *J. Exp. Bot.* 63 4919–4929. 10.1093/jxb/ers173 22791833 PMC3428009

[B38] Wise M. J. Tunnacliffe A. (2004). POPP the question: what do LEA proteins do? *Trends Plant Sci.* 9 13–17. 10.1016/j.tplants.2003.10.012 14729214

[B39] Xie C. Zhang R. X. Qu Y. T. Miao Z. Y. Zhang Y. Q. Shen X. Y. (2012). Overexpression of MtCAS31 enhances drought tolerance in transgenic Arabidopsis by reducing stomatal density. *New Phytol.* 195 124–135. 10.1111/j.1469-8137.2012.04136.x 22510066

[B40] Yang W. Zhang L. Lv H. Li H. Zhang Y. Xu Y. (2015). The K-segments of wheat dehydrin WZY2 are essential for its protective functions under temperature stress. *Front. Plant Sci.* 6:406 10.3389/fpls.2015.00406ref PMC4467595 26124763

